# The combined antibacterial effects of sodium new houttuyfonate and berberine chloride against growing and persistent methicillin-resistant and vancomycin-intermediate *Staphylococcus aureus*

**DOI:** 10.1186/s12866-020-02003-2

**Published:** 2020-10-19

**Authors:** Xue Li, Penghe Wang, Xinxin Hu, Youwen Zhang, Xi Lu, Congran Li, Tongying Nie, Guoqing Li, Xiukun Wang, Jing Pang, Yun Lu, Xinyi Yang, Xuefu You

**Affiliations:** 1 Beijing Key Laboratory of Antimicrobial Agents, Institute of Medicinal Biotechnology, Chinese Academy of Medical Sciences & Peking Union Medical College, Beijing, 100050 China; 2 Wuya College of Innovation, Shenyang Pharmaceutical University, Shenyang, 110016 China

**Keywords:** Combination therapy, Berberine, Sodium new houttuyfonate, Persistence, MRSA, VISA

## Abstract

**Background:**

Infections caused by drug-resistant *Staphylococcus aureus*, especially vancomycin-intermediate *Staphylococcus aureus* (VISA), leave clinicians with limited therapeutic options for treatment. Persister cells is a leading cause of recalcitrant infection and antibiotic treatment failure, and there is no drug in clinical use that specifically targets persister cells currently. Here, we report a promising combination therapy of sodium new houttuyfonate (SNH) and berberine chloride (BBR) which is able to eradicate both growing and persistent drug-resistant *Staphylococcus aureus.*

**Results:**

The susceptibility test showed SNH exhibited anti-MRSA activity with MIC_90_ at 64 μg/mL, while BBR showed weak anti-MRSA activity with MIC_90_ at 512 μg/mL. MICs of BBR in combination with 1/2 MIC SNH decreased by 4 to 64 folds compared with MICs of BBR alone. The results of time-killing assays revealed that the combined use of sub-MIC SNH and BBR offered an in vitro synergistic action against growing MRSA (including pathogenic MRSA) and VISA strains. More importantly, the combination of SNH and BBR was able to eradicate VISA Mu50 and pathogenic MRSA persister cells. The synergistic effect is likely related to the interruption of the cell membrane caused by SNH, which is confirmed by scanning electron microscope and membrane potential and permeability analysis.

**Conclusions:**

Our study provide a promising clinical curative strategy for combating drug-resistant *S. aureus* infections, especially for recalcitrant infections caused by persister cells.

**Supplementary information:**

**Supplementary information** accompanies this paper at 10.1186/s12866-020-02003-2.

## Background

*Staphylococcus aureus* is one of the most common Gram-positive human pathogens that cause both superficial and invasive infections [1, 2]. Over the past two decades, methicillin-resistant *S. aureus* (MRSA) with multidrug resistance has become increasingly prevalent in both health-care and community settings, and glycopeptide antibiotics such as vancomycin have been used as the last line of defense against MRSA infections [3–5]. However, with the widespread use of glycopeptide antibiotics, MRSA strains with reduced susceptibility to vancomycin such as vancomycin-intermediate *S. aureus* (VISA) have also emerged [6, 7]. In addition to the development of antibiotic resistance, there is increasing concern on persister cells of *S. aureus* as they are suspected to be a leading cause of chronic and refractory infections [8, 9]. Persister cells are a subpopulation that exhibits transiently high antibiotic tolerance while the majority of the bacterial population are killed [9]. They are often slow-growing or growth-arrested and able to resume growth when the stress is removed, which can lead to recalcitrant infection and antibiotic treatment failure [10–13]. Due to the burden arising from antibiotic resistance and persister formation in *S. aureus*, new antibiotics and therapeutic options are urgently needed.

Berberine, an isoquinoline alkaloid originally obtained from plants such as *Phellodendri chinensis* Cortex and *Coptis chinensis* Franch and fully chemically synthesized now [14], has been widely used to treat bacterial diarrhea and gastroenteritis for many years in China [15, 16]. Several studies have demonstrated the antibacterial activities of berberine alone or as a synergist of antibiotics [17–22]. However, the antibacterial activity of berberine used alone is weak, and none of these studies have revealed its ability to inhibit persister cells. This motivates us to investigate on BBR-based combination therapy against drug-resistant and/or persistent *S. aureus*.

Previously we discovered that sodium new houttuyfonate (SNH) showed synergistic effect against *S. aureus* when combined with oxacillin or netilmicin [23], which attracted our interest in investigating it in the BBR-based combination therapy. SNH (C_14_H_27_NaO_5_S, MW = 330.4) is a chemically stable derivative of houttuynin which is an active ingredient from a traditional Chinese herb *Houttuynia cordata* Thunb [24]. Studies have shown that houttuynin exhibits a variety of medicinal properties including activities on anti-inflammatory, anti-viral and antibacterial [25–27]. However, the bioactivities of SNH such as the antibacterial effect are poorly investigated.

Here we report a promising synergistic combination of SNH and berberine chloride (BBR) which is likely in use for the treatment of MRSA and VISA infections, especially recalcitrant infections caused by persister cells. In this study, we systematically evaluated the in vitro activity of SNH and BBR, alone and in combination, against MRSA and VISA and for the first time found their strong synergistic effect on both growing and persister cells. The action of SNH with BBR on the cell membrane was examined to investigate the underlying mechanism of synergism.

## Results

### Antibacterial spectrum of SNH and BBR against gram-positive and -negative pathogens

The results of antibacterial spectrum assay (Table 1) showed that SNH was active against Gram-positive bacteria including *staphylococci* and *enterococci* with MICs ranging from 16 to 64 μg/mL, while BBR exhibited poor antibacterial activity for all tested Gram-positive bacteria with MIC ranging from 8 to > 128 μg/mL. Both SNH and BBR showed little antibacterial activity against the tested Gram-negative bacteria with MIC higher than 128 μg/mL.

**Table 1:** Antibacterial spectrum of SNH and BBR

Bacterial species	Strains	MIC (μg/mL)	
		SNH	BBR
**Gram-positive**			
*Staphylococcus epidermidis*	ATCC12228	32	> 128
	CCPM(A)-P-021301	16	32
	CCPM(A)-P-021303	16	8
*Staphylococcus aureus*	ATCC29213	32	128
	ATCC33591	64	> 128
	CCPM(A)-P-011315	32	> 128
	CCPM(A)-P-011317	32	64
	CCPM(A)-P-011318	32	128
*Enterococcus faecalis*	ATCC29212	32	> 128
	ATCC51299	16	> 128
	ATCC51575	16	> 128
	CCPM(A)-P-051304	64	> 128
**Gram-negative**			
*Escherichia coli*	ATCC25922	> 128	> 128
	CCPM(A)-P-071515	> 128	> 128
	CCPM(A)-P-071410	> 128	> 128
	CCPM(A)-P-071411	> 128	> 128
*Klebsiella pneumoniae*	ATCC700603	> 128	> 128
	ATCCBAA2146	> 128	> 128
	CCPM(A)-P-080007	> 128	> 128
	CCPM(A)-P-081404	> 128	> 128
	CCPM(A)-P-081415	> 128	> 128
*Pseudomonas aeruginosa*	ATCC27853	> 128	> 128
	PA01	> 128	> 128
	CCPM(A)-P-091346	> 128	> 128
*Acinetobacter baumannii*	ATCC19606	> 128	> 128
*Enterobacter cloacae*	ATCC43560	> 128	> 128
*Enterobacter aerogenes*	ATCC13048	> 128	> 128
*Serratia marcescens*	ATCC31074	> 128	> 128
*Providencia rettgeri*	ATCC31052	> 128	> 128
*Proteus mirabilis*	CCPM(A)-P-131301	> 128	> 128
*Stenotrophomonas maltophilia*	ATCC13636	> 128	> 128
*Citrobacter freundii*	ATCC43864	> 128	> 128

### MICs of BBR and SNH against *S. aureus* strains

**Fig. 1:**
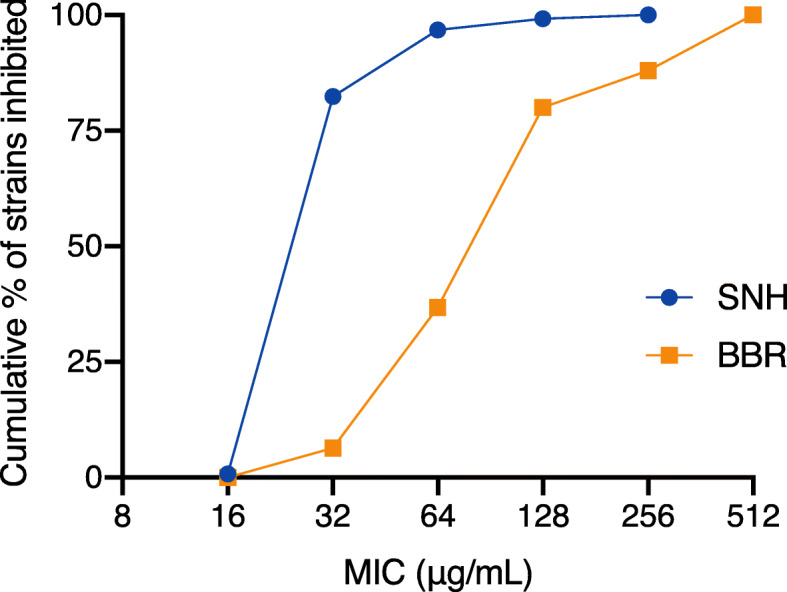
Cumulative inhibitory rate (%) of MRSA strains at different MIC levels of SNH and BBR

The MICs of SNH and BBR against 124 strains of MRSA are shown in Table 2 and the cumulative inhibition of the bacteria at different MIC levels of the two compounds was presented in Fig. 1. SNH inhibited all the MRSA strains at concentrations ranging from 16 to 256 μg/mL, with MIC_50_ and MIC_90_ values at 32 and 64 μg/mL, respectively, which are essentially consistent with previous reports [23]. BBR showed moderate activity against *S. aureus* and the MIC range of BBR for the tested MRSA strains was 32-512 μg/mL, with MIC_50_ and MIC_90_ at 128 and 512 μg/mL. The MICs of SNH and BBR for the quality control strain ATCC 29213 were 32 and 128 μg/mL, respectively, all within the expected ranges as reported previously [23, 28, 29].

**Table 2:** Susceptibility of MRSA strains to SNH and BBR

Organism	Number of stains	Antimicrobial agent	MIC (μg/mL)		
			Range	50%	90%
MRSA	124	SNH	16-256	32	64
		BBR	32-512	128	512

### MICs of BBR against *S. aureus* in combination with sub-MICs of SNH

**Fig. 2:**
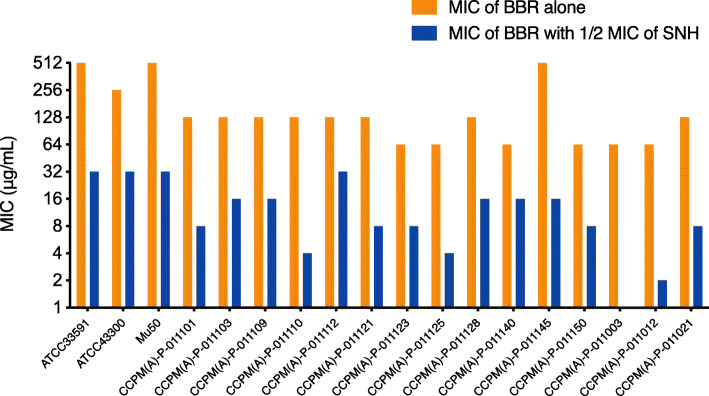
MICs of BBR alone and in combination with 1/2 MIC SNH

To examine the antibacterial effect of BBR combined with sub-MIC level of SNH, 15 MRSA clinical isolates were randomly selected with three ATCC strains being used as controls. The MICs of BBR alone and in combination with 1/2 MIC of SNH were presented in Table 3 and Fig. 2. MICs of BBR decreased by 4 to 64 folds in combination with 1/2 MIC of SNH when compared with MICs of BBR alone.

**Table 3:** MIC of BBR alone and in combination with 1/2 MIC SNH against MRSA strains

Strains	MIC alone (μg/mL)		BBR with 1/2 MIC of SNH	
	SNH	BBR	MIC (μg/mL)	Fold decreased
ATCC33591	64	512	32	16
ATCC43300	64	256	32	16
Mu50	64	512	32	16
CCPM(A)-P-011101	32	128	8	16
CCPM(A)-P-011103	32	128	16	8
CCPM(A)-P-011109	32	128	16	8
CCPM(A)-P-011110	32	128	4	32
CCPM(A)-P-011112	32	128	32	4
CCPM(A)-P-011121	32	128	8	16
CCPM(A)-P-011123	32	64	8	8
CCPM(A)-P-011125	32	64	4	16
CCPM(A)-P-011128	32	128	16	8
CCPM(A)-P-011140	32	64	16	4
CCPM(A)-P-011145	64	512	16	32
CCPM(A)-P-011150	32	64	8	8
CCPM(A)-P-011003	32	64	1	64
CCPM(A)-P-011012	64	64	2	32
CCPM(A)-P-011021	64	128	8	16

### Time-killing assay of SNH-BBR combination on growing MRSA and VISA cells

**Fig. 3:**
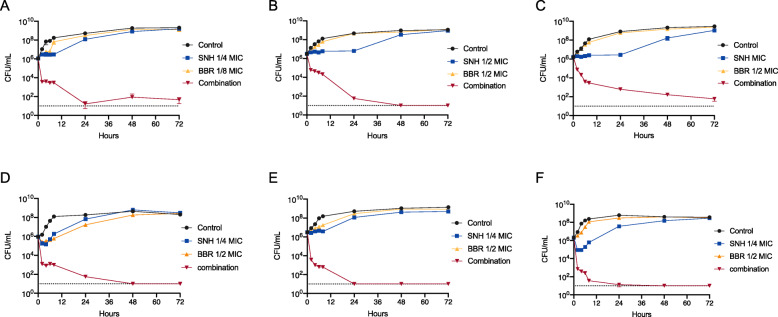
The combination of SNH and BBR killed growing MRSA and VISA cells. **a** MRSA ATCC33591 treated with SNH (16 μg/mL, 1/4 MIC), BBR (64 μg/mL, 1/8MIC) or their combination. **b** MRSA ATCC43300 treated with SNH (32 μg/mL, 1/2 MIC), BBR (128 μg/mL, 1/2MIC) or their combination. **c** VISA Mu50 treated with SNH (64 μg/mL, MIC), BBR (256 μg/mL, 1/2MIC) or their combination. **d** MRSA CCPM(A)-P-0116173 treated with SNH (8 μg/mL, 1/4 MIC), BBR (64 μg/mL, 1/2MIC) or their combination. **e** MRSA CCPM(A)-P-010850 treated with SNH (8 μg/mL, 1/4 MIC), BBR (32 μg/mL, 1/2MIC) or their combination. **f** MRSA CCPM(A)-P-011012 treated with SNH (16 μg/mL, 1/4 MIC), BBR (32 μg/mL, 1/2MIC) or their combination. The lower limit of detection is indicated by a dotted line

Time-killing assays were performed with MRSA ATCC33591, ATCC43300, VISA Mu50 and three pathogenic MRSA clinical isolates CCPM(A)-P-0116173, CCPM(A)-P-010850 and CCPM(A)-P-011012. As shown in Fig. 3, SNH alone inhibited the growth of the tested bacteria within 8-24 h despite the subsequent regrowth of the bacteria, while BBR alone had little effect on bacterial growth. In contrast, the combination of SNH and BBR resulted in significant synergistic effect on all the tested strains and exhibited a typical bactericidal mode of action. The SNH-BBR combinations eradicated the viable count by ≥4log10 CFU/mL when compared with the starting inoculum or with any of the most active agent used alone, with the viable counts below the detection limit (10 CFU/mL) for ATCC33591, ATCC43300, CCPM(A)-P-0116173, CCPM(A)-P-010850 and CCPM(A)-P-011012. The synergistic effect was observed as early as 2 h after SNH-BBR treatment and lasted for 72 h.

### The combination of SNH and BBR eradicated VISA and pathogenic MRSA persister cells

Keren et al. has shown that almost all stationary phase *S. aureus* cells are persistent and exhibited tolerance to antibiotics [30]. To validate that the stationary phase cells used in our study are persistent, MIC determination and killing assay of ciprofloxacin and linezolid against strains used in the persister assays were performed. The stationary phase cells of ciprofloxacin-susceptible MRSA strain ATCC33591 exhibited tolerance to ciprofloxacin (10 μg/mL) (Table S1, Figure S1A). The stationary phase cells of all the strains were tolerant to linezolid (Figure S1B) although they are susceptible to linezolid based on the MIC values.

**Fig. 4:**
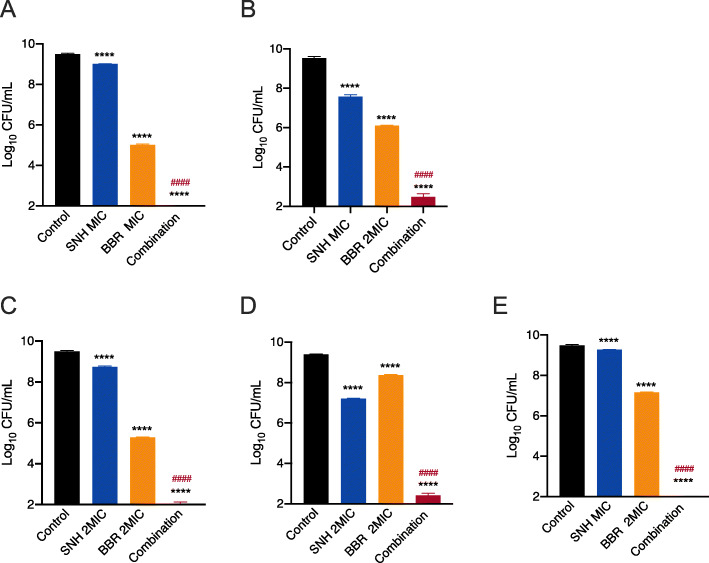
The combination of SNH and BBR eradicated *S. aureus* persister cells. **a** MRSA ATCC33591 persister cells treated with SNH (64 μg/mL, MIC), BBR (512 μg/mL, MIC) or SNH-BBR combination for 24 h. **b** VISA Mu50 persister cells treated with SNH (64 μg/mL, MIC), BBR (1024 μg/mL, 2MIC) or SNH-BBR combination for 24 h. **c** Clinical MRSA CCPM(A)-P-0116173 persister cells treated with SNH (64 μg/mL, 2MIC), BBR (256 μg/mL, 2MIC) or SNH-BBR combination for 24 h. **d** Clinical MRSA CCPM(A)-P-010850 treated with SNH (64 μg/mL, 2MIC), BBR (128 μg/mL, 2MIC) or SNH-BBR combination for 24 h. **e** Clinical MRSA CCPM(A)-P-01012 treated with SNH (64 μg/mL, MIC), BBR (128 μg/mL, 2MIC) or SNH-BBR combination for 24 h. The y-axis starts at the value of the minimum detection limit. Asterisks (*) denote statistical significance compared with untreated control, while hash marks (#) indicate significant differences between the groups of SNH-BBR combination and BBR alone, as determined by one-way ANOVA followed by Turkey’s multiple-comparison test (****, *P* < 0.0001; ####, *P* < 0.0001)

To evaluate the effectiveness of SNH and BBR against persister cells, ATCC33591 (MRSA), Mu50 (VISA) and three pathogenic MRSA CCPM(A)-P-0116173, CCPM(A)-P-010850 and CCPM(A)-P-011012 were cultured with shaking for 24 h to reach stationary phase. As shown in Fig. 4, compared with the untreated control, treatment with SNH or BBR alone significantly decreased the number of persistent *S. aureus*, with a greater reduction of persister cells in the BBR treatment group (except for CCPM(A)-P-010850). When compared with any agent used alone, the SNH-BBR combination reduced the number of *S. aureus* persisters by at least 3log10, and persister cells of ATCC33591 and pathogenic MRSA CCPM(A)-P-0116173 and CCPM(A)-P-011012 were totally eradicated by the combination (Fig. 4a, c and e).

### Effects of the SNH and BBR on cellular morphology

**Fig. 5:**
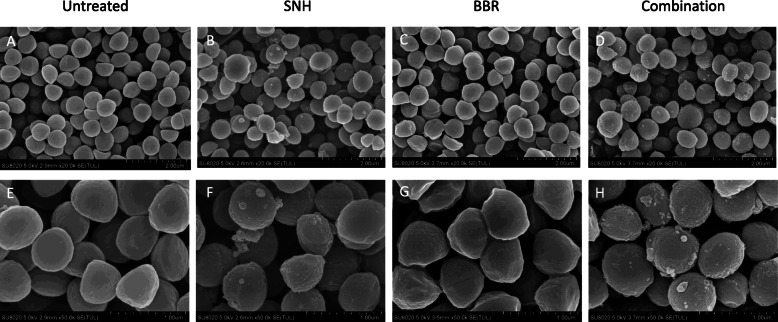
Effects of SNH and BBR on *S. aureus* ATCC33591 using SEM analysis. The images were acquired at a magnification of 20,000 (upper panel, **a-d**) or 50,000 (bottom panel, **e-h**) times. The bacterial cells were treated with nothing (**a**, **e**), 1/4MIC of SNH (**b**, **f**), 1/8MIC of BBR (**c**, **g**) or combination of 1/4MIC SNH and 1/8MIC BBR (**d**, **h**) for 4 h incubation

The structure of SNH contains a hydrophilic sulfinyl head and a hydrophobic alkyl tail with 12 carbon atoms, indicating amphiphilic properties [23]. Our hypothesis for the synergistic effect between SNH and BBR is that SNH may disrupt the cell membrane and facilitated the entrance of BBR into the bacterial cell. To address this, we examined the cell morphology of *S. aureus* ATCC33591 treated with sub-MIC concentration of SNH or BBR by scanning electron microscope (Fig. 5). Cells of ATCC33591 or BBR-treated ATCC33591 exhibited a relatively “clean” surface. However, bacterial cells treated with SNH or SNH-BBR combination showed a “rough” cell surface with granules on it, which may be the result of leakage of cell contents.

### SNH increased the permeability and decreased the membrane potential

If SNH acts as a surfactant and causes damage on *S. aureus* cell membrane, changes in the permeability and membrane potential of the bacteria should be observed after treatment with the compound. To determine the effect of SNH and BBR on the membrane potential and permeability, fluorescent dyes 3,3-DiOC_2_(3) and TO-PRO-3 were used as quantitative indicators, respectively. The red/green fluorescence ratio of DiOC_2_(3) provides a cell size-independent measure of bacterial membrane potential. TO-PRO-3 is believed to be membrane impermeant and the far-red fluorescence of the dye (~ 695 nm) can be detected with increased cell permeability. CCCP reduces cell membrane potential to zero but does not affect permeability, while Nisin reduces membrane potential and increases membrane permeability as well. They were selected as a positive control for cell membrane potential and permeability detection, respectively. The concentrations used for SNH (1/4 MIC) and BBR (1/8 MIC) were based on the time-killing assay.

**Fig. 6:**
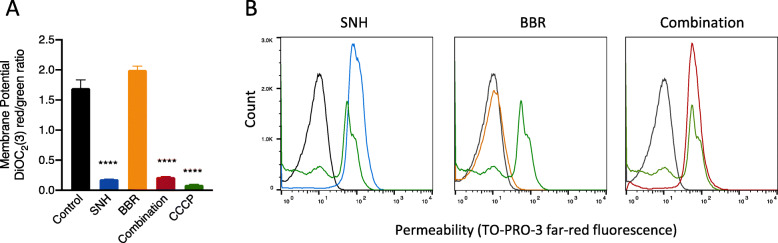
SNH led to a decreased potential and an increased permeability of cell membrane in *S. aureus* ATCC33591. Bacterial membrane potential (**a**) was represented by the ratio of red/green fluorescence of DiOC_2_(3). CCCP (5 μM) was used as the positive control in the DiOC_2_(3)-based membrane potential assay. Bacterial membrane permeability (**b**) was measured by TO-PRO-3. Nisin (25 μg/mL, green) was used as a positive control in the TO-PRO-3-based membrane permeability assay. Untreated ATCC33591 cells were regarded as a negative control (black) in both assays. Cells treated with 1/4 MIC SNH, 1/8 MIC BBR and the SNH-BBR combination were presented as blue, orange and red, respectively. Asterisks denote statistical significance as determined by one-way ANOVA followed by Tukey’s multiple-comparison analysis ****, *P* < 0.0001

As shown in Fig. 6, sub-MIC of SNH reduced the membrane potential (Fig. 6a) and increased the membrane permeability of the bacterial cell (Fig. 6b) to the same extent as the positive controls (CCCP and nisin, respectively). However, BBR had no effect on the membrane potential and permeability of the bacteria. The cells treated with sub-MIC BBR exhibited the same properties as the untreated cells. The combination of sub-MIC SNH and BBR led to a decreased membrane potential and an increased membrane permeability of the MRSA strain as the same as SNH alone. The results firmly supported our hypothesis that SNH, not BBR, interrupts the cell membrane integrity.

## Discussion

The current limited extent of new drug development is being massively outpaced by emerging antibiotic resistance, pushing humans to the edge of a “post-antibiotic” age. Despite many efforts on drug discovery, very limited new classes of antibiotics have been developed for clinical uses during the past decades. The growing need to combat antimicrobial resistance calls for developing new effective strategies. Combinations of antibiotics or antimicrobial agents offer a productive strategy to deal with the global threat of increasing resistance [31].

Berberine has been part of traditional Chinese medicine for thousands of years with extensive bioactivities such as antimicrobial activities [32]. In this study, the combination of BBR and SNH showed a synergistic effect against antibiotic-resistant *S. aureus*. The in vitro antibacterial activities of the two compounds were evaluated against 121 clinical MRSA isolates, as well as ATCC strains. To maximize the relevance of our research to clinical situations, the clinical isolates covered almost all types of clinical infections, including samples from nasal colonization, superficial skin and soft tissue infection, and blood infection (Table S1). BBR showed poor antibacterial activity against *S. aureus* with MIC_90_ at 512 μg/mL, which is consistent with previous studies and might be attributed to the strong hydrophilicity and low permeability through cell membrane due to the quaternary ammonium group of BBR [18, 19]. Therefore, the combined use of compounds that increase membrane permeability (such as SNH) with BBR may increase the amount of the latter into bacterial cells and promote antibacterial activity. As predicted, the combination of sub-MIC SNH and BBR in this study eradicated growing MRSA cells to the limit of detection as shown in the time-killing assay. Notably, the combination therapy is not only effective on MRSA strains, but on VISA strain Mu50. This is of great significance since there are few options for clinicians on treating VISA infections.

It is well recognized now that recalcitrant infections are related to the survival of dormant persister cells. However, there is no drug in clinical use that specifically targets persister cells currently. In this study, we found that sub-MIC levels of SNH or BBR can significantly reduce the number of *S. aureus* persister cells and the combination of the two compounds effectively eradicated the persisters including MRSA and VISA strains (Fig. 4). Surprisingly, the ability of killing pathogenic MRSA persister cells was exhibited at sub-MIC of SNH and BBR (Figure S2). Treatment with 1/2 MIC of SNH in combination with 1/2 MIC BBR decreased 3log10 of CFU/mL of pathogenic *S. aureus* persister cells compared with any other groups. Kim et al. [33] demonstrated that antimicrobial agents inducing rapid permeabilization of cell membrane should be effective against MRSA persisters. In our study, we found that SNH decreased the membrane potential to the same extent of CCCP, and increased cell permeability considerably (Fig. 6). Considering that membrane-active agents may exhibit toxicity in mammalian cells [34], hemolytic activities of SNH and BBR were tested against human erythrocytes (Figure S3). The hemolysis assay showed that SNH caused approximately 6% hemolysis at MIC_90_ and did not cause hemolysis at MIC_50_, and BBR had no hemolytic effect.

In view of the morphological observations and the analysis of membrane potential and permeability in *S. aureus* in the present study, the synergistic effect between BBR and SNH could be caused by SNH interrupting the cell membrane thus promoting BBR into microbial cells. Previous studies proposed a strategy of the combination of BBR with multidrug-resistance pump inhibitors against *S. aureus* [35, 36]. Our work raises the possibility that combination of BBR with antimicrobial agents that targeting the cell membrane is also an effective strategy against drug-resistant *S. aureus*. Notably, the concentration of BBR used in the combination therapy is relatively high compared with commonly used antibiotics, which might limit its application on systematic administration. However, reduction of concentration of BBR can be achieved by structural modification, and several berberine derivates with better antibacterial activity have been reported recently [37, 38]. Moreover, several studies had put efforts into developing transdermal products of BBR to deliver the drug safely and efficiently [39, 40]. Besides, BBR has been used topically for the treatment of burns, acne and periodontal inflammation in clinical trials [41–43]. Considering that *S. aureus* is the leading cause of skin and soft tissue infections and topical creams are more acceptable for patients with skin infections, the topical products of SNH-BBR combination are of great clinical significance in the future. Further studies will focus on determining the in vivo efficacy of this combination therapy in rodent models and screening more antimicrobial agents targeting bacterial cell membrane to expand the list of BBR-based combination therapy.

## Conclusions

A small portion of microbial persisters can cause recurrent or intractable infections, eventually increasing resistance to antibiotics. Infections caused by drug-resistant *S. aureus*, especially VISA, leave clinicians with few therapeutic options for treatment. In the present study, we demonstrated the synergistic antibacterial effect of SNH and BBR in killing growing and persistent MRSA and VISA cells. Our study also provides a promising combination strategy for combating drug-resistant *S. aureus* infections, especially for recalcitrant infections caused by persister cells.

## Methods

### Bacterial strains

A total of 121 MRSA strains from hospitals in China between 2008 and 2017 were employed in this study. All bacterial strains used throughout this study were obtained from the Collection Center of Pathogen Microorganism of Chinese Academy of Medical Sciences (CAMS-CCPM) in China. All clinical isolates identified as MRSA were confirmed with the VITEK 2-COMPACT system (bioMerieux, Marcy I’Etoile, France), the standard oxacillin agar dilution method recommended by the Clinical and Laboratory Standards Institute (CLSI) [44] and detection of *mecA* gene by PCR (primers are Forward, 5′- TCCAGATTACAACTTCACCAGG-3′, and Reverse, 5′-CCACTTCATATCTTGTAACG-3′). Relevant characteristics of the clinical isolates of MRSA utilized in this study are presented in Table S1. *S. aureus* strains from the American Type Culture Collection (ATCC), ATCC29213 (MSSA), ATCC33591 (MRSA), ATCC43300 (MRSA) and/or Mu50 (ATCC700699, VISA) were used as quality control strains in this study according to the needs of specific assays. All isolates were stored at − 80 °C and streaked on tryptic soy agar (TSA) plates to make overnight cultures.

### Antimicrobial agents

Sodium new houttuyfonate (SNH) was provided by Beijing Standard Herbs Medical Science & Technology Development Co. LTD (Beijing, China). Berberine chloride (BBR) was commercially purchased from Haoyang Biotech Co. LTD. (Shaanxi, China). SNH was dissolved in warmed distilled water as described previously [23]. BBR was dissolved in DMSO as a stock solution at a concentration of 10.24 mg/mL and stored at − 20 °C after sterilizing through 0.22 μm filters.

### Susceptibility test

The antibacterial spectrum of SNH and BBR was tested with the agar dilution method according to CLSI [44]. Basically, serial two-fold dilutions of SNH or BBR ranging from 128 to 0.03 μg/mL were made in 15 mL sterile Mueller–Hinton (MH) agar. All isolates including Gram-positive and -negative bacterial strains were tested at a final inoculum of 10^4^ CFU per spot on MH agar with serial dilutions of SNH or BBR using a multipoint inoculator (Denley Instruments) and incubated at 35 °C for 24 h. The minimum concentration with no bacterial growth was considered to be the MIC of the compound.

The MICs of SNH and BBR against *S. aureus* were further determined by the broth microdilution method according to CLSI [44]. Briefly, 100 μL of serially diluted compounds (starting concentrations were 1024 μg/mL) in cation-adjusted MH (CAMH) broth were added to wells of 96-well plates. Ten microliter of 5 × 10^6^ CFU/mL log phase bacterial culture was added to each well. The plates were incubated at 35 °C for 24 h and the MIC values were determined by the minimal concentration of wells that with the absence of visual turbidity as read with the naked eye.

Combination antimicrobial susceptibility test was performed based on MIC assay with only small modification. Briefly, serial-diluted BBR in combination with SNH at a final concentration of 1/2 MIC was filled in the wells of a 96-well plate and the bacteria were inoculated at a final turbidity of 5 × 10^5^ CFU/mL in each well. After incubation of the plate at 35 °C for 24 h, the MIC values of BBR combined with 1/2 MIC SNH were read.

All the MIC values (single or in combination) were determined in triplicate on different days.

### Time-killing assays

Kinetics of bactericidal activity of SNH and BBR alone and in combination were determined by time-killing assay according to the protocol published previously [23]. The experiment was performed against ATCC33591, ATCC43300, Mu50 and three randomly selected MRSA clinical isolate CCPM(A)-P-0116173, CCPM(A)-P-010850 and CCPM(A)-P-011012. Briefly, overnight cultures were diluted 1:1000 in fresh medium and grown to a turbidity of OD_600nm_ = 0.8, then diluted to 10^6^ CFU/mL with CAMH broth in a 250 mL sterile flask. Sub-MIC concentrations of SNH and BBR alone or in combination were added. At specified time intervals (0, 2, 4, 6, 8, 24, 48 and 72 h), 100 μL aliquots were serially diluted with 10-fold in 0.9% saline, plated on TSA plates in triplicates and incubated at 35 °C for 24 h. Then the viable colonies were counted after the incubation. The combination of SNH and BBR was considered synergistic if the bacterial killing showed a ≥ 2log10 decrease in colony count as compared to the most active monotherapy.

### Persister assay

MRSA ATCC33591, VISA Mu50 and three pathogenic MRSA isolates CCPM(A)-P-0116173, CCPM(A)-P-010850 and CCPM(A)-P-011012 were tested for killing of persisters by antimicrobials. Previous work has shown that almost all stationary phase *S. aureus* cells are tolerant to conventional antibiotics and persistently behaving, thus being used in persister assay [30, 45]. To validate the persister cells used in our study are tolerant to antibiotics, an overnight culture of *S. aureus* strains was diluted 1:1000 and cultured at 35 °C, 220 rpm for 3 h (ATCC33591) or 24 h (all the strains) to reach log phase or stationary phase respectively. One milliliter of log phase or stationary phase cells were challenged with 10 μg/mL ciprofloxacin (for ATCC33591) or 20 μg/mL linezolid (all the strains) for 3 h at 35 °C. Before and after the antibiotic challenge a sample was serial diluted and viable cells were counted.

Colonies from a fresh plate were inoculated into 10 mL of tryptic soy broth (TSB) and grown at 35 °C, 220 rpm for 24 h to form a persister phenotype in stationary phase. The starting inoculum was about 10^9^ CFU/mL for each strain. Appropriate concentrations of SNH and BBR, alone or in combination, were added to each tube with *S. aureus* persister cells. At specified time points, 10 μL aliquots of samples were removed from the tubes, serially diluted and spot-plated onto TSA plates to determine CFU/mL.

### Scanning electron microscopy

An overnight culture of *S. aureus* ATCC33591 was diluted 1:1000 in TSB and grown to log phase (OD_600nm_ = 0.8) at 35 °C. Bacterial cells were then incubated with or without sub-MIC level of SNH, BBR or their combination for 4 h, and collected and fixed with 2.5% glutaraldehyde overnight. The fixed bacterial cells were washed and resuspended in phosphate buffer. The samples were dehydrated, gold-coated and observed by scanning electron microscope (Hitachi SU8020, Japan).

### Bacterial membrane potential and permeability determinations

The membrane potential and permeability of bacteria were determined using dyes of diethyloxacarbocyanine iodide (DiOC_2_(3)) and TO-PRO-3, respectively, as previously reported [45, 46]. Overnight culture of *S. aureus* ATCC33591 was diluted to approximately 10^6^ CFU/mL and treated with sub-MIC of SNH, BBR or their combination for 4 h at 37 °C. Parallelly, carbonyl cyanide 3-chlorophenylhydrazone (CCCP, 5 μM) treated cells were used as a positive control to depolarize bacterial membrane potential by eradicating the proton gradient, while untreated cells were set as the negative control. Nisin (25 μg/mL) reduces membrane potential and renders bacterial cells permeable to TO-PRO-3, and was selected as the positive control for detecting cell permeability.

The cells were then stained with 30 μM DiOC_2_(3) and 100 nM TO-PRO-3 (Molecular Probes, USA) for 15 min at room temperature. After centrifugation, washing and resuspension, 1 mL of stained cells were pipetted for analysis on a BD FACSCalibur flow cytometer and 100,000 counts were recorded. DiOC_2_(3) was excited at 488 nm, and its green and red fluorescence were detected through a 525- and 610-nm bandpass filter, respectively. Red fluorescence is emitted from DiOC_2_(3) aggregates, which depends on the size and membrane potential of bacteria cells, while green fluorescence generated from a single dye molecule varies with the size of cells or clumps, and is largely independent of bacterial membrane potential. Thus, the red/green fluorescence ratio provides a cell size-independent measure of bacterial membrane potential. TO-PRO-3 was excited at 630 nm, and its far-red fluorescence was detected through a 695-nm long-pass emission filter. TO-PRO-3 with positive charges can enter and stain nucleic acids to produce substantially increased fluorescence in bacteria with damaged membranes, but excluded by bacteria with intact cell membranes. The dye is therefore used to indicate the permeability of bacterial cell membranes.

### Statistical analysis

Statistical analysis was performed with GraphPad Prism 8 software. *P*-values were calculated by one-way ANOVA followed by Tukey’s multiple comparisons test to analyze the differences between each pair of groups. *P*-values of < 0.05 were considered significant. Each experiment was performed at least 3 times and presented as mean ± SD.

## Supplementary information

**Additional file 1: Figure S1.** Stationary phase cells of *S. aureus* were tolerant to antibiotics, **Figure S2.** MRSA persisters were killed by sub-MIC level of SNH-BBR combination, **Figure S3.** Hemolytic activity of SNH and BBR against human erythrocytes, **Table S1.** MIC of ciprofloxacin and linezolid against *S. aureus* strains used in persister assay, **Table S2.** Information of clinical strains used in this study.

## Data Availability

The datasets during and/or analysed during the current study available from the corresponding author on reasonable request.

## Abbreviations

*S. aureus*: *Staphylococcus aureus*
MRSA: Methicillin-resistant *S. aureus*
VISA: Vancomycin-intermediate *S. aureus*
SNH: Sodium new houttuyfonate
BBR: Berberine chloride
CAMS-CCPM: Collection Center of Pathogen Microorganism of Chinese Academy of Medical Sciences
CLSI: Clinical and Laboratory Standards Institute
ATCC: American Type Culture Collection
MH: Mueller–Hinton
CAMH: Cation-adjusted Mueller–Hinton
MIC: Minimum inhibitory concentration
TSB: Tryptic soy broth
CFU: Colony-forming unit
CCCP: Carbonyl cyanide 3-chlorophenylhydrazone
DiOC_2_(3): Diethyloxacarbocyanine iodide

## References

[1] Lowy FD (1998). Staphylococcus aureus infections. N Engl J Med.

[2] Kennedy AD (2008). Epidemic community-associated methicillin-resistant Staphylococcus aureus: recent clonal expansion and diversification. Proc Natl Acad Sci.

[3] David MZ, Daum RS (2010). Community-associated methicillin-resistant Staphylococcus aureus: epidemiology and clinical consequences of an emerging epidemic. Clin Microbiol Rev.

[4] Chambers HF, DeLeo FR (2009). Waves of resistance: Staphylococcus aureus in the antibiotic era. Nat Rev Microbiol.

[5] Foster TJ (2017). Antibiotic resistance in Staphylococcus aureus. Current status and future prospects. FEMS Microbiol Rev.

[6] Howden BP (2010). Reduced vancomycin susceptibility in Staphylococcus aureus, including vancomycin-intermediate and heterogeneous vancomycin-intermediate strains: resistance mechanisms, laboratory detection, and clinical implications. Clin Microbiol Rev.

[7] McGuinness WA, Malachowa N, DeLeo FR (2017). Focus: infectious diseases: vancomycin resistance in Staphylococcus aureus. Yale J Biol Med.

[8] Conlon BP (2016). Persister formation in Staphylococcus aureus is associated with ATP depletion. Nat Microbiol.

[9] Wang Y (2018). Inactivation of TCA cycle enhances Staphylococcus aureus persister cell formation in stationary phase. Sci Rep.

[10] Harms A, Maisonneuve E, Gerdes K (2016). Mechanisms of bacterial persistence during stress and antibiotic exposure. Science.

[11] Fisher RA, Gollan B, Helaine S. Persistent bacterial infections and persister cells. Nat Rev Microbiol. 2017;15(8):453–64. 10.1038/nrmicro.2017.42 28529326

[12] Brauner A, et al. Distinguishing between resistance, tolerance and persistence to antibiotic treatment. Nat Rev Microbiol. 2016;14(5):320–30. 10.1038/nrmicro.2016.34 27080241

[13] Balaban NQ (2004). Bacterial persistence as a phenotypic switch. Science.

[14] Iwasa K (1998). Antimicrobial activity of 8-alkyl-and 8-phenyl-substituted berberines and their 12-bromo derivatives. J Nat Prod.

[15] Feng R (2015). Transforming berberine into its intestine-absorbable form by the gut microbiota. Sci Rep.

[16] Chen C (2015). A randomized clinical trial of berberine hydrochloride in patients with diarrhea-predominant irritable bowel syndrome. Phytother Res.

[17] Guo N (2015). The synergy of berberine chloride and totarol against Staphylococcus aureus grown in planktonic and biofilm cultures. J Med Microbiol.

[18] Tan J (2019). Antimicrobial characteristics of Berberine against prosthetic joint infection-related Staphylococcus aureus of different multi-locus sequence types. BMC Complement Altern Med.

[19] Cui H (2018). Preparation and evaluation of Antidiabetic agents of Berberine organic acid salts for enhancing the bioavailability. Molecules.

[20] Liang R-m (2014). Potent in vitro synergism of fusidic acid (FA) and berberine chloride (BBR) against clinical isolates of methicillin-resistant Staphylococcus aureus (MRSA). World J Microbiol Biotechnol.

[21] Yu H-H (2005). Antimicrobial activity of berberine alone and in combination with ampicillin or oxacillin against methicillin-resistant Staphylococcus aureus. J Med Food.

[22] Zuo G-Y (2012). Antibacterial and synergy of berberines with antibacterial agents against clinical multi-drug resistant isolates of methicillin-resistant Staphylococcus aureus (MRSA). Molecules.

[23] Lu X, et al. In vitro activity of sodium new houttuyfonate alone and in combination with oxacillin or netilmicin against methicillin-resistant Staphylococcus aureus. PLoS One. 2013;8(7):e68053. 10.1371/journal.pone.0068053. 10.1371/journal.pone.0068053 PMC3699466 23844154

[24] Sodium new houttuyfonate (2016). ACS Chemistry for Life.

[25] Lu H (2006). Variation in chemical composition and antibacterial activities of essential oils from two species of Houttuynia T HUNB. Chem Pharm Bull.

[26] Lu H (2006). Anti-inflammatory effect of Houttuynia cordata injection. J Ethnopharmacol.

[27] Lau K-M (2008). Immunomodulatory and anti-SARS activities of Houttuynia cordata. J Ethnopharmacol.

[28] Endo E, Dias Filho B (2015). Antibacterial activity of berberine against methicillin-resistant Staphylococcus aureus planktonic and biofilm cells. Austin J Trop Med Hyg.

[29] Guo J-J (2016). The anti-Staphylococcus aureus activity of the phenanthrene fraction from fibrous roots of Bletilla striata. BMC Complement Altern Med.

[30] Keren I (2004). Persister cells and tolerance to antimicrobials. FEMS Microbiol Lett.

[31] Tyers M, Wright GD (2019). Drug combinations: a strategy to extend the life of antibiotics in the 21st century. Nat Rev Microbiol.

[32] Imenshahidi M, Hosseinzadeh H (2016). Berberis vulgaris and berberine: an update review. Phytother Res.

[33] Kim W (2015). Identification of an antimicrobial agent effective against methicillin-resistant Staphylococcus aureus Persisters using a fluorescence-based screening strategy. PLoS One.

[34] Hurdle JG (2011). Targeting bacterial membrane function: an underexploited mechanism for treating persistent infections. Nat Rev Microbiol.

[35] Stermitz FR (2000). Synergy in a medicinal plant: antimicrobial action of berberine potentiated by 5′-methoxyhydnocarpin, a multidrug pump inhibitor. Proc Natl Acad Sci.

[36] Stermitz FR (2002). Two flavonols from Artemisa annua which potentiate the activity of berberine and norfloxacin against a resistant strain of Staphylococcus aureus. Planta Med.

[37] Wang J (2017). The synthesis and antistaphylococcal activity of 9, 13-disubstituted berberine derivatives. Eur J Med Chem.

[38] Fan TY (2018). Synthesis and antibacterial evaluation of 13-substituted cycloberberine derivatives as a novel class of anti-MRSA agents. Eur J Med Chem.

[39] Patel NAPRP, Patel RKPNJ. The formulation and evaluation of topical berberine-hydrochloride products. Pharm Technol. 2010;34(1). https://www.pharmtech.com/view/formulation-and-evaluation-topical-berberine-hydrochloride-products.

[40] Buchanan B, et al. Comparative pharmacokinetics and safety assessment of transdermal berberine and dihydroberberine. PLoS One. 2018;13(3):e0194979. 10.1371/journal.pone.0194979. 10.1371/journal.pone.0194979 PMC5868852 29579096

[41] Kapoor S, Saraf S (2011). Topical herbal therapies an alternative and complementary choice to combat acne. Res J Med Plant.

[42] Taghavi AM (2012). Effect of Berberine gel on periodontal inflammation: clinical and histological. J Periodontol.

[43] See G-J (2001). Evaluating the role of alternative therapy in burn wound management: randomized trial comparing moist exposed burn ointment with conventional methods in the management of patients with conventional methods in the management of patients with second-degree burns. MedGenMed.

[44] Institute, C.a.L.S (2018). Performance standards for antimicrobial susceptibility testing.

[45] Allison KR, Brynildsen MP, Collins JJ (2011). Metabolite-enabled eradication of bacterial persisters by aminoglycosides. Nature.

[46] Shapiro HM, Nebe-von-Caron G. Multiparameter flow cytometry of bacteria, Methods Mol Biol. 2004;263:33-44. 10.1385/1-59259-773-4:033. 10.1385/1-59259-773-4:033 14976359

