# Supplementary material for: The combined antibacterial effects of sodium new houttuyfonate and berberine chloride against growing and persistent methicillin-resistant and vancomycin-intermediate Staphylococcus aureus
Source: BMC Microbiol. 2020 Oct 19;20:317. doi: 10.1186/s12866-020-02003-2 (PMC7574187; doi:10.1186/s12866-020-02003-2)
Supplement: Supplementary file 1 — Additional file 1: Figure S1. Stationary phase cells of S. aureus were tolerant to antibiotics, Figure S2. MRSA persisters were killed by sub-MIC level of SNH-BBR combination, Figure S3. Hemolytic activity of SNH and BBR against human erythrocytes, Table S1. MIC of ciprofloxacin and linezolid against S. aureus strains used in persister assay, Table S2. Information of clinical strains used in this study. [file 12866_2020_2003_MOESM1_ESM.pdf]

Supplements.

1. Methods for Hemolytic assay

Hemolytic activity of SNH and BBR on human erythrocytes was performed using a previously published protocol [1]. In a 96-well microtiter plate, 100 µL of 4% human erythrocytes was added to 100 µl of serially diluted compound. 0.2% DMSO and 2% Triton-X 100 were used as a negative and a positive control, respectively. After incubation for 1 h at 37°C, the 96-well plate was centrifuged at 500 g for 5 min. 100 µL of the supernatant from each well was carefully transferred to a fresh microtiter plate, and the absorbance of supernatants was measured at 540 nm. Percent hemolysis was calculated as $(A_{sample} - A_{0.1\%\ DMSO}) / (A_{1\%\ Triton\ X\text{-}100} - A_{sample}) \times 100$.

2. Supporting Figures

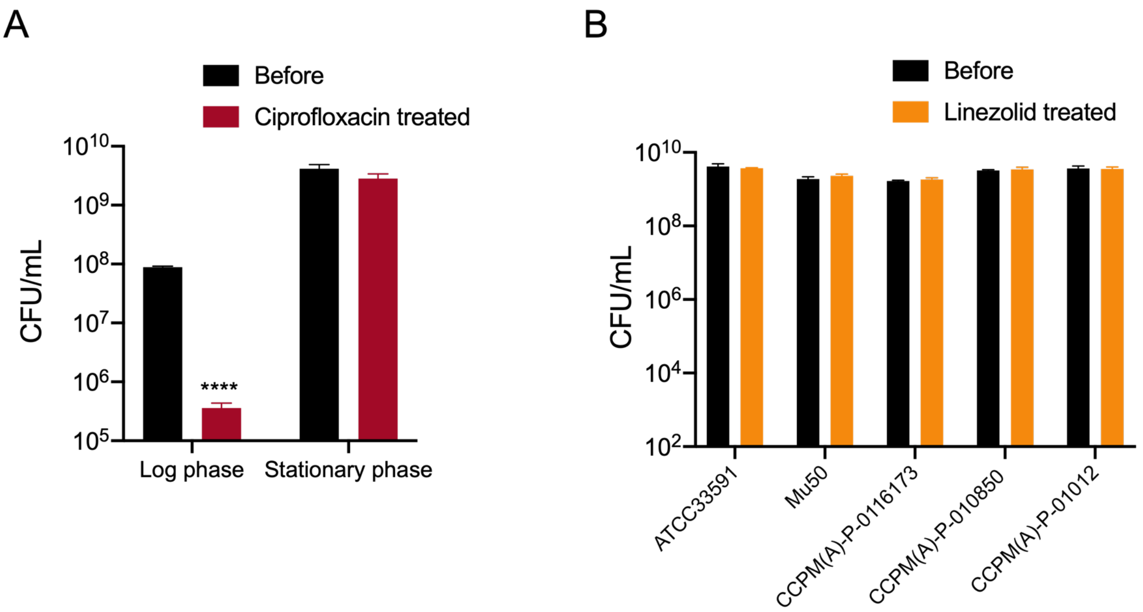

Figure S1. Stationary phase cells of *S. aureus* were tolerant to antibiotics. (A) Log phase and stationary phase cells of ATCC33591 were challenged with ciprofloxacin (10 µg/mL) for 3h. Cell count before (black) and after (red) challenge were recorded. (B) Stationary phase cells of *S. aureus* strains were challenged with linezolid (20 µg/mL) for 3h. Cell count before (black) and after (orange) challenge were recorded. Asterisks denote statistical significance as determined by t test (****, $P < 0.0001$)

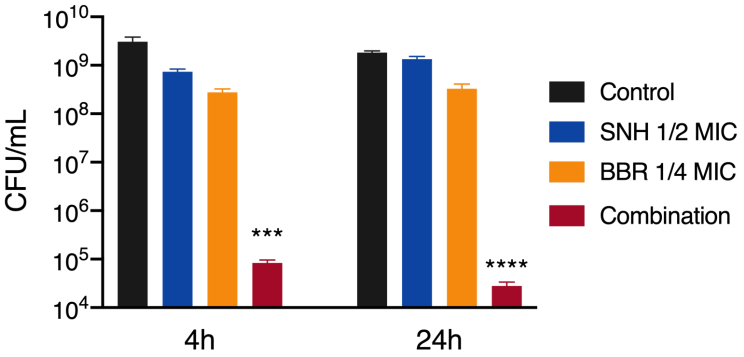

Figure S2. MRSA persisters were killed by sub-MIC level of SNH-BBR combination. Pathogenic MRSA CCPM(A)-P-0116173 after treatment with nothing (control), SNH (16 µg/mL, 1/2MIC), BBR (64 µg/mL, 1/2 MIC) or their combination for 4 h and 24 h. Asterisks denote statistical significance as determined by one-way ANOVA followed by Tukey's multiple-comparison analysis (***, $P < 0.001$; ****, $P < 0.0001$)

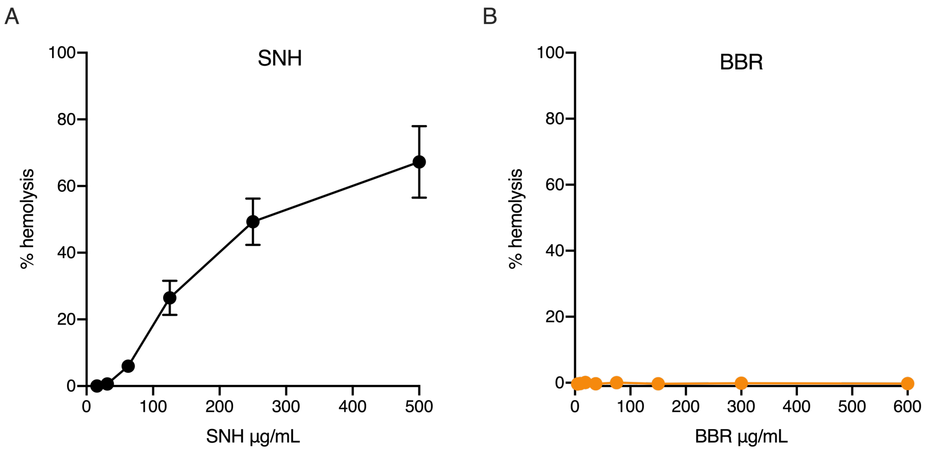

Figure S3. Hemolytic activity of SNH and BBR against human erythrocytes. 4% human erythrocytes were treated with serial two-fold dilutions of SNH (A) or BBR (B) for 1 h at 37°C. A sample treated with 1% Triton-X 100 was used as the control for 100% hemolysis. Results are shown as means ± SD; n=3.

3. Supporting Table

Table S1. MIC of ciprofloxacin and linezolid against *S. aureus* strains used in persister assay.

Strain	MIC (µg/mL)	
	Ciprofloxacin	Linezolid
ATCC 33591	≤0.5 (S*)	2 (S)
Mu50	≥ 16 (R)	≤0.25 (S)
CCPM(A)-P-0116173	≥ 16 (R)	≤0.25 (S)
CCPM(A)-P-010850	≥ 16 (R)	≤0.25 (S)

CCPM(A)-P-011012	≥ 16 (R)	2 (S)

* S, susceptible; R, resistant. According to CLSI MIC breakpoints for *S. aureus* (30th edition) [2]

Table S2. Information of clinical strains used in this study.

Strains	Detection of MRSA			Origins #
	Oxacillin R* (μg/mL)	Cefoxitin R** (VITEK 2)	*mecA*	
CCPM(A)-P-010821	> 120	POS	+	ND
CCPM(A)-P-010850	> 120	POS	+	ND
CCPM(A)-P-010911	> 120	POS	+	PE
CCPM(A)-P-010913	> 120	POS	+	SP
CCPM(A)-P-010916	> 120	POS	+	SP
CCPM(A)-P-010920	> 120	POS	+	SP
CCPM(A)-P-010921	> 120	POS	+	SP
CCPM(A)-P-010925	> 120	POS	+	NPS
CCPM(A)-P-010927	> 120	POS	+	NPS
CCPM(A)-P-010928	> 120	POS	+	NA
CCPM(A)-P-010931	> 120	POS	+	SP
CCPM(A)-P-010933	> 120	POS	+	SP
CCPM(A)-P-011001	> 120	POS	+	NA
CCPM(A)-P-011002	> 120	POS	+	NA
CCPM(A)-P-011003	> 120	POS	+	NA
CCPM(A)-P-011005	> 120	POS	+	NA
CCPM(A)-P-011006	> 120	POS	+	NA
CCPM(A)-P-011007	> 120	POS	+	NA
CCPM(A)-P-011008	> 120	POS	+	NA
CCPM(A)-P-011009	> 6	POS	+	NA
CCPM(A)-P-011012	> 120	POS	+	NA
CCPM(A)-P-011013	> 120	POS	+	NA
CCPM(A)-P-011014	> 120	POS	+	NA
CCPM(A)-P-011016	> 120	POS	+	NA
CCPM(A)-P-011017	> 120	POS	+	NA
CCPM(A)-P-011018	> 120	POS	+	NA
CCPM(A)-P-011020	> 120	POS	+	NA
CCPM(A)-P-011021	> 120	POS	+	NA
CCPM(A)-P-011022	> 120	POS	+	NA
CCPM(A)-P-011024	> 120	POS	+	B
CCPM(A)-P-011025	> 6	POS	+	SP
CCPM(A)-P-011026	> 6	POS	+	B
CCPM(A)-P-011028	> 120	POS	+	WD
CCPM(A)-P-011029	> 120	POS	+	SP
CCPM(A)-P-011030	> 120	POS	+	SP
CCPM(A)-P-011031	> 6	POS	+	NA

CCPM(A)-P-011032	> 120	POS	+	SP
CCPM(A)-P-011033	> 120	POS	+	B
CCPM(A)-P-011034	> 120	POS	+	SP
CCPM(A)-P-011036	> 120	POS	+	SP
CCPM(A)-P-011038	> 120	POS	+	B
CCPM(A)-P-011039	> 6	POS	+	SP
CCPM(A)-P-011040	> 120	POS	+	B
CCPM(A)-P-011041	> 120	POS	+	B
CCPM(A)-P-011042	> 120	POS	+	SP
CCPM(A)-P-011043	> 120	POS	+	SP
CCPM(A)-P-011044	> 120	POS	+	B
CCPM(A)-P-011046	> 120	POS	+	SP
CCPM(A)-P-011047	> 120	POS	+	AS
CCPM(A)-P-011048	> 120	POS	+	WD
CCPM(A)-P-011049	> 120	POS	+	SP
CCPM(A)-P-011050	> 120	POS	+	WD
CCPM(A)-P-011051	> 6	POS	+	B
CCPM(A)-P-011052	> 120	POS	+	DVCT
CCPM(A)-P-011053	> 6	POS	+	AB
CCPM(A)-P-011054	> 120	POS	+	SP
CCPM(A)-P-011056	> 120	POS	+	DVCT
CCPM(A)-P-011057	> 120	POS	+	DVCT
CCPM(A)-P-011058	> 120	POS	+	B
CCPM(A)-P-011060	> 120	POS	+	SP
CCPM(A)-P-011061	> 120	POS	+	NA
CCPM(A)-P-011062	> 120	POS	+	B
CCPM(A)-P-011063	> 120	POS	+	SP
CCPM(A)-P-011064	> 120	POS	+	SP
CCPM(A)-P-011065	> 120	POS	+	SP
CCPM(A)-P-011066	> 6	POS	+	SP
CCPM(A)-P-011067	> 120	POS	+	NA
CCPM(A)-P-011068	> 120	POS	+	B
CCPM(A)-P-011071	> 120	POS	+	SP
CCPM(A)-P-011072	> 6	POS	+	SP
CCPM(A)-P-011076	> 120	POS	+	NA
CCPM(A)-P-011101	> 120	POS	+	SP
CCPM(A)-P-011102	> 120	POS	+	SP
CCPM(A)-P-011103	> 120	POS	+	SP
CCPM(A)-P-011104	> 120	POS	+	SP
CCPM(A)-P-011105	> 120	POS	+	SP
CCPM(A)-P-011106	> 120	POS	+	SP
CCPM(A)-P-011107	> 120	POS	+	SP
CCPM(A)-P-011108	> 120	POS	+	SP

CCPM(A)-P-011109	> 120	POS	+	SP
CCPM(A)-P-011110	> 120	POS	+	SP
CCPM(A)-P-011112	> 120	POS	+	SP
CCPM(A)-P-011113	> 120	POS	+	SP
CCPM(A)-P-011115	> 120	POS	+	SP
CCPM(A)-P-011116	> 120	POS	+	SP
CCPM(A)-P-011119	> 120	POS	+	DI
CCPM(A)-P-011120	> 120	POS	+	SP
CCPM(A)-P-011121	> 120	POS	+	SP
CCPM(A)-P-011122	> 120	POS	+	DI
CCPM(A)-P-011123	> 120	POS	+	SP
CCPM(A)-P-011124	> 120	POS	+	SP
CCPM(A)-P-011125	> 120	POS	+	SP
CCPM(A)-P-011126	> 120	POS	+	SP
CCPM(A)-P-011127	> 120	POS	+	SP
CCPM(A)-P-011128	> 120	POS	+	SP
CCPM(A)-P-011130	> 120	POS	+	SP
CCPM(A)-P-011131	> 120	POS	+	SP
CCPM(A)-P-011132	> 120	POS	+	SP
CCPM(A)-P-011135	> 120	POS	+	SP
CCPM(A)-P-011136	> 120	POS	+	SP
CCPM(A)-P-011137	> 120	POS	+	SP
CCPM(A)-P-011138	> 120	POS	+	SP
CCPM(A)-P-011139	> 120	POS	+	SP
CCPM(A)-P-011140	> 120	POS	+	SP
CCPM(A)-P-011143	> 120	POS	+	SP
CCPM(A)-P-011144	> 120	POS	+	SP
CCPM(A)-P-011145	> 120	POS	+	SP
CCPM(A)-P-011147	> 120	POS	+	NPS
CCPM(A)-P-011148	> 120	POS	+	SP
CCPM(A)-P-011150	> 120	POS	+	SP
CCPM(A)-P-011151	> 120	POS	+	SP
CCPM(A)-P-011152	> 120	POS	+	SP
CCPM(A)-P-011154	> 120	POS	+	SP
CCPM(A)-P-011157	> 120	POS	+	CSF
CCPM(A)-P-0116144	> 6	POS	+	B
CCPM(A)-P-0116167	> 120	POS	+	U
CCPM(A)-P-0116173	> 120	POS	+	B
CCPM(A)-P-011744	> 120	POS	+	PE
CCPM(A)-P-011754	> 6	POS	+	DI

* Oxacillin^{R}: Oxacillin resistance using oxacillin agar dilution. All strains were tested against 6 and 120 µg/mL oxacillin. According to CLSI, growth on 6 µg/mL plate reported as MRSA strains.

** Cefoxitin^{R}: Cefoxitin resistance tested by VITEK 2 using AST-GP67 Susceptibility Card containing 6 μg/mL cefoxitin, "POS" represent resistance to cefoxitin, "+" means the PCR test of mecA being positive.

Abbreviations: SP: sputum, DI: discharge, NPS: nasopharyngeal swab, CSF: cerebrospinal fluid, B: blood, AS: ascites, WD: wound discharge, AB: abscess, DVCT: deep venous catheter tip, PE: pleural effusion, PICC: PICC catheter, BAL: bronchial-alveolar lavage, U: urine, ND: nose discharge.

References

1. Kim, W., et al., NH125 kills methicillin-resistant *Staphylococcus aureus* persisters by lipid bilayer disruption. Future medicinal chemistry, 2016. 8(3): p. 257-269.
2. Institute, C.a.L.S., *Performance standards for antimicrobial susceptibility testing*, 30ed. CLSI supplement M100. **2020**
